# Efficiency of GnRH–Loaded Chitosan Nanoparticles for Inducing LH Secretion and Fertile Ovulations in Protocols for Artificial Insemination in Rabbit Does

**DOI:** 10.3390/ani11020440

**Published:** 2021-02-08

**Authors:** Eman M. Hassanein, Nesrein M. Hashem, Kheir El-Din M. El-Azrak, Antonio Gonzalez-Bulnes, Gamal A. Hassan, Mohamed H. Salem

**Affiliations:** 1 Animal and Fish Production Department, Faculty of Agriculture, Alexandria University, Alexandria 21545, Egypt; eman.mostafa1177@yahoo.com (E.M.H.); kheir_elazrak@yahoo.com (K.E.-D.M.E.-A.); gamaleldeen.hassan@alexu.edu.eg (G.A.H.); mhsalem15@yahoo.com (M.H.S.); 2 Departamento de Reproduccion Animal, INIA, Avda. Puerta de Hierro s/n., 28040 Madrid, Spain; 3 Departamento de Produccion y Sanidad Animal, Facultad de Veterinaria, Universidad Cardenal Herrera-CEU, CEU Universities, C/ Tirant lo Blanc, 7, 46115 Alfara del Patriarca, Valencia, Spain

**Keywords:** artificial-insemination, GnRH, nanotechnology, ovulation, rabbit

## Abstract

**Simple Summary:**

Nano-drug delivery systems can be employed for improving ovulation induction prior to artificial insemination (AI) in rabbits. In this study, different routes of administration and different doses of GnRH–loaded chitosan nanoparticles (GnRH–ChNPs) were assessed for inducing ovulation in rabbits, proving their usefulness to reduce the GnRH dose and animal handling and improving AI outcomes. The use of GnRH–ChNPs allows for the reduction of the conventional intramuscular GnRH dose to half without compromising fertility. However, the addition of GnRH–ChNPs to semen extenders, although successfully inducing ovulation, has negative impacts on fertility. Thus, more studies are needed to allow further adjustments.

**Abstract:**

Gonadotropin-releasing hormone (GnRH)–loaded chitosan nanoparticles (GnRH–ChNPs) were used at different doses and routes of administration to induce ovulation in rabbits as an attempt to improve artificial insemination (AI) procedures and outcomes. In this study, the characteristics (size, polydispersity, loading efficiency, and zeta-potential) of GnRH–ChNPs and the GnRH release pattern were determined in an in vitro study. A first in vivo study assessed the pituitary and ovarian response to different GnRH–ChNPs doses and routes of administration (two i.m. doses, Group HM = 0.4 µg and Group QM = 0.2 µg, and two intravaginal doses, Group HV = 4 µg and Group QV = 2 µg) against a control group (C) receiving bare GnRH (0.8 µg). The HM, QM, and HV treatments induced an earlier LH-surge (90 min) than that observed in group C (120 min), whilst the QV treatment failed to induce such LH surge. The number of ovulation points was similar among treatments, except for the QV treatment (no ovulation points). A second in vivo study was consequently developed to determine the hormonal (progesterone, P_4_, and estradiol, E_2_) profile and pregnancy outcomes of both HM and HV treatments against group C. The treatment HM, but not the treatment HV, showed adequate P_4_ and E_2_ concentrations, conception and parturition rates, litter size, litter weight, and viability rate at birth. Overall, the use of GnRH–ChNPs allows for a reduction in the conventional intramuscular GnRH dose to half without compromising fertility. However, the addition of GnRH–ChNPs to semen extenders, although successfully inducing ovulation, has negative impacts on fertility. Thus, more studies are needed to explore this point and allow further adjustments.

## 1. Introduction

The occurrence of external stimulation during mating (neurohormonal reflex) is required to evoke the gonadotropin-releasing hormone (GnRH)-dependent luteinizing hormone (LH) surge, and consequently ovulation, in non-spontaneously ovulating species like the rabbit [1]. This biological event plays a crucial limiting role for the successful application of assisted reproductive techniques such as artificial insemination (AI) because, in the absence of the male, ovulation needs to be induced by artificial hormonal stimulation. Hence, the induction of ovulation in routine AI protocols is usually achieved by the administration of either GnRH or LH or other gonadotrophins with LH activity (i.e., equine chorionic gonadotrophin, eCG, or human chorionic gonadotrophin, hCG). The GnRH analogues (such as gonadorelin, lecirelin, triptorelin, or buserelin) are the most recommended compounds because they have additional biological advantages and the possibility of repeated treatments without developing specific antibodies [2].

Currently, GnRH is included in semen extenders to be directly administered to the doe via the seminal dose, which improves animal management and welfare [2]. Certainly, the successful implementation of the intravaginal route in protocols for ovulation induction may minimize animal distress and staff workload when compared to traditional protocols via intramuscular doses. However, the enzymatic proteolytic activity of seminal plasma and vaginal fluids and the biological barriers for mucosal permeation may limit the efficiency of such protocols.

In addition, GnRH has a short half-life time in blood circulation, 2–4 min, because it is rapidly degraded by peptidases and cleared by glomerular filtration [3], which limits both its biological activity and sustained action. Consequently, a first approach for improving the efficiency of the treatment is to increase the dose or the activity of the GnRH analogue. However, doses more than ten to fifteen-fold higher are needed (about 8 and 16 µg buserelin/seminal dose), which constitutes a potential health risk for workers and significantly increases the cost-efficiency of the protocols [4].

A promising alternative may be based on the use of nano-drug delivery systems. Nano-drug delivery systems have been optimized to extend the half-life time of the compound to improve its passage across endothelial or epithelial barriers into blood or lymph circulation and to sustain its delivery to the target sites. Consequently, overcoming these biological barriers can improve cellular uptake and therefore lower doses of drugs/hormones may be used [5,6,7,8,9]. Hence, nano-drug systems for hormonal treatments (e.g., GnRH for ovulation induction) may benefit many biotechnological assisted reproductive techniques in the livestock field (e.g., AI [5]). However, there is a scarcity of data regarding both hormone bioactivity and animal performance when GnRH is used in nano-formula, although previous results are promising. To date, chitosan-dextran sulfate GnRH (buserelin acetate) nanoparticles have been added to seminal doses of rabbits [10], allowing the GnRH dose to be reduced to half the conventional dose without affecting the fertility of the does. Moreover, the results of a trial performed with the administration of chitosan-tripolyphosphate (TPP)-conjugated GnRH nanoparticles in goats indicate that the GnRH dose may be even reduced three- to four-fold without affecting fertility and prolificacy [11].

The practical application of such protocols makes necessary extensive studies on technical and physiological features that may limit the efficiency and final outcomes of treatments involving GnRH-loaded nanoparticles (fabrication, route of administration, bioavailability and hormonal balance). Therefore, the present study aimed to set the basis and evaluate the feasibility and efficiency of GnRH nanoparticles for inducing ovulation in rabbits. Three consecutive experiments (first an in vitro approach and afterward, two in vivo trials) were performed to (1) manufacture and evaluate the physicochemical properties and release patterns of GnRH-loaded chitosan nanoparticles, and (2) determine the ovarian response, hormonal balance, and reproductive performance of does after ovulation induction with different doses and administration routes (intramuscular vs. intravaginal) of such GnRH nanoparticles.

## 2. Materials and Methods

### 2.1. Manufacturing of Gonadotropin-Releasing Hormone (GnRH)-Loaded Chitosan Nanoparticles

Chitosan (chitosan extra pure; with a degree of deacetylation >85%; molecular weight = 300–350 KD; Alpha Chemika, Mubia, India) and sodium tripolyphosphate (TPP; Thermo Fisher GmbH, Kandel, Germany) were used in the fabrication of a nano-carrier polymer following the steps of the ionic gelation method [10,11,12]. Briefly, chitosan (0.1%, wt/vol) was vigorously stirred in an aqueous acidic solution (1%, wt/vol) to obtain polymeric chitosan cations. An aqueous solution of TPP (0.1 g/dL) was also prepared. Afterward, the chitosan-TPP nanoparticles were prepared by slowly dropping the TPP solution into a chitosan solution (chitosan to TPP weight ratio was 2:1) under constant magnetic stirring (800 rpm) for 2 h at room temperature. The pH of the chitosan-TPP solution was adjusted to 5.5 and then stored in the refrigerator before exposure to any analysis or application. GnRH–loaded chitosan nanoparticles (GnRH–ChNPs) were prepared by dripping-wise the hormone (GnRH; Receptal^®^, MSD, Intervet International GmbH, Unterschleißheim, Germany) solution to the fabricated ChNPs in a ratio of 1:1. The mixture was adjusted to a pH of 6.5 and after that stirred gently at 800 rpm for 60 min at room temperature and incubated overnight to allow the hormonal adsorption on the surface of the nanoparticles.

### 2.2. In Vitro Assessment of the Physicochemical Properties and Release Patterns of GnRH-Loaded Nanoparticles

Particle size, zeta potential, and size distribution (polydispersity index, PdI) of the free ChNPs and GnRH–ChNPs were measured using a Zetasizer instrument (Malvern Instruments, Malvern, UK), which is based on dynamic light scattering (DLS) techniques. The samples were analyzed in triplicate and the average value (±SD) of each parameter was calculated. The functional groups of chitosan (Ch), ChNPs, and GnRH–ChNPs were identified by Fourier transform infrared spectrophotometer (FTIR, a Perkins Elmer 1600, USA) in the range from 4000 to 400/cm using potassium bromide discs (KBr) (5 mg of particles, 100 mg KBr pellets). Morphological characteristics of the free ChNPs and GnRH–ChNPs were examined using transmission electron microscope (TEM; JEOL JEM-1400, 120 kV, Peabody, MA, USA). Freshly fabricated ChNPs and GnRH–ChNPs solutions were diluted with deionized water with adjusted pH close to neutral. A one-drop sample was placed on a carbon-coated film 300 mesh copper grid and was left for 10 min until air-dried. The sample was stained with 1 M uranyl acetate solution for 1.5 min at 7 °C and any excess uranyl acetate was removed with filter paper before viewing on the TEM at magnification × 20,000.

The hormonal loading efficiency (LE%) was determined by the separation of the nanoparticles from the aqueous medium by centrifugation at 1200× *g* at 4 °C for 20 min. The amount of free hormone in the supernatant of GnRH–ChNPs solution was measured using UV spectrophotometry at a wavelength of 280 nm (Optizen Pop, Mecasys Co. Ltd., Daejeon, Korea) and using the supernatant of ChNPs solution as a blank [13]. Afterward, LE% was calculated as ([initial GnRH concentration − free GnRH concentration]/initial GnRH concentration × 100).

The assessment of release patterns was carried out for 48 h to plot the kinetic release profile of GnRH from ChNPs according to the method described by Dounighi and co-workers [13]. In brief, a pre-weighed sample of GnRH-ChNPs was divided into three aliquots and dissolved in equal volumes of phosphate buffer solution (PBS; pH 7.4). The samples were then incubated at 37 °C in a shaker adjusted at 600 rpm for 48 h. At allocated time intervals (0.5, 1, 2, 4, 6, 10, 12, 24, 48 h), the samples were removed and centrifuged at 14,000 rpm and 4 °C for 20 min. The concentration of free GnRH released into the supernatant was evaluated by spectrophotometry at the 280 nm wavelength (Optizen pop, Mecasys Co. Ltd. Daejeon, Korea). The amount of GnRH was calculated using non-loaded nanoparticles as a blank and then LE% was determined as previously described.

### 2.3. In vivo Assessment of the Reproductive Response of Does Treated with GnRH–Loaded Nanoparticles

#### 2.3.1. Animals and Ethic Statement

A total of 62 female rabbits were used. The experiments were carried out at the Laboratory of Rabbit Physiology Research, Faculty of Agriculture, Alexandria University, Egypt (31°20′N, 30°E). The does were individually housed in standard wire cage batteries (40 × 50 × 35 cm) at the same rabbitry (10.97 ± 0.36 h daylight length, 19.85 ± 1.03 °C temperature and 74.92 ± 2.543% relative humidity). Does were fed ad-libitum with a pelleted diet, containing 17.73% crude protein and 68.96% TDN) and had free access to water. All animals were handled according to the principles of animal care published by the European Union Directive 2010/63/UE on the protection of animals used for research and, in agreement, the experimental procedures used were previously assessed and approved by the INIA Committee of Ethics in Animal Research (report CEEA2014/087).

#### 2.3.2. Trial 1. Patterns of LH Secretion, Ovulatory Efficiency, and Early Fertility

**Figure 1:**
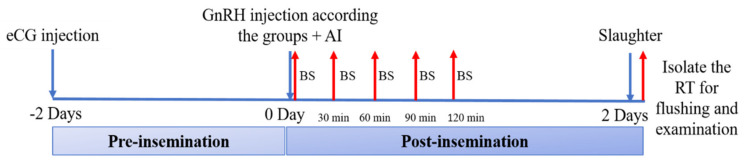
Experimental planning of trial 1, aiming to determine the effect of gonadotrophin-releasing hormone (GnRH) in different doses and routes of administration on LH secretion (by means of successive blood samples; BS) and presence of oocytes/embryos in the reproductive tract, RT, at day 2 after artificial insemination (AI).

Twenty adult nulliparous rabbit does (5–5.5 months-old and 2.82 ± 0.21 kg of body-weight) were treated for estrus synchronization with 25 IU of eCG (Gonaser^®^, Hipra, Girona, Spain) [14] and 48 h later, does were subjected to one of the following five ovulation induction treatments: a control conventional i.m. protocol (0.8 µg of GnRH in distilled water; C-treatment) and four protocols using GnRH–ChNPs with half i.m. dose (0.4 µg. GnRH–ChNPs; HM-treatment), quarter i.m. dose (0.2 µg i.m. GnRH–ChNPs; QM-treatment), half vaginal dose (4 µg vs. GnRH–ChNPs; HV-treatment), and quarter vaginal dose (2 µg vs. GnRH–ChNPs; QV-treatment). Females in C, HM, and HV treatments were immediately inseminated artificially with 0.3 mL diluted semen (30 × 10^6^ sperm/insemination). Females in HV and QV treatments were inseminated, at the same time, using the same seminal dose, but supplemented with the corresponding GnRH–ChNPs. Semen samples used for AI were collected from five fertile rabbit bucks by using an artificial vagina and a teaser doe. The quality of semen was evaluated, and only semen samples fulfilling quality criteria were used for AI [15]. The doses of GnRH–ChNPs were based on the findings of previous studies, where the dose of 0.8 µg GnRH is the conventional dose in intramuscular treatment and dose of 8 µg GnRH is the conventional dose in intravaginal treatment [4,11]. The schematic diagram of the experiment is presented in Figure 1.

The assessment of the patterns of LH secretion was performed on blood samples collected from the marginal ear vein of each doe with non-heparinized tubes at 0 (time of insemination) 30, 60, 90, and 120 min post-insemination. The samples were centrifuged at 700× *g* for 20 min to obtain serum, which was stored at −20 °C until analysis. Then, the serum LH concentration was determined using an enzyme immunoassay commercial kit (Cusabio Biotech, Hubei, China). The assay sensitivity was 0.35 mIU/mL and intra- and inter-assay coefficients of variation were 8% and 10%, respectively.

Afterward, all females were sacrificed 48 h post-insemination and the reproductive tracts were immediately obtained. The ovaries were dissected for determining the number of total ovarian follicles (clear visible follicles) and the number of ovulatory points (ovulated follicles), as previously described [14]. The oviducts were flushed with 15 mL of phosphate buffer solution (PBS) containing 0.2% of bovine serum albumin (BSA, Sigma-Aldrich, Madrid, Spain) to assess the number of embryos and their development stage and the number of non-fertilized oocytes with a binocular microscope (Olympus Optical Co. Ltd., Tokyo, Japan).

#### 2.3.3. Trial 2. Fertility and Productive Traits

**Figure 2:**
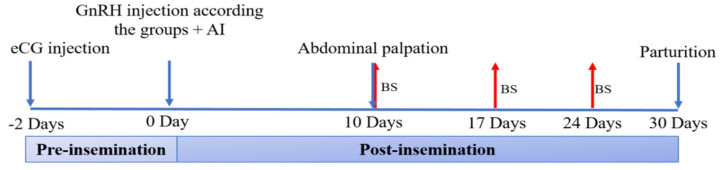
Experimental planning of trial 2, aiming to determine the effect of gonadotrophin-releasing hormone (GnRH) in different routes of administration on hormonal patterns (by means of successive blood samples; BS) and fertility and reproductive traits after artificial insemination (AI).

Forty-two adult nulliparous rabbit does (5–5.5 months-old and 2.41 ± 0.21 kg of body-weight) were treated for ovulation induction and AI as described in trial 1 but, according to the results of such experiment, only three treatments were used (C, HM, and HV; *n* = 14/treatment). The schematic diagram of the experiment is presented in Figure 2.

Pregnancy diagnosis was performed by abdominal palpation at Day 10 after AI. Blood samples were collected at days 10, 17, and 24 of pregnancy, as previously described, to determine the concentrations of progesterone (P_4_) and estradiol (E_2_) by using commercial enzyme immunoassay kits (Monobind Inc. Lake Forest, California, CA, USA.). The assay sensitivities were 0.105 ng/mL for P_4_ and 8.2 pg/mL for E_2_, while intra- and inter-assay coefficients of variation were 8.4% and 7.6% for P_4_ and 8.6% and <5.6% for E_2_.

Fertility and productive traits were recorded in terms of conception rate ([number of pregnant does on Day 10/total number of inseminated does] × 100), parturition rate ([number of delivering does/total number of inseminated does] × 100), abortion rate ([number of aborted does/number of pregnant does] × 100), litter size ([number of kits at birth/number of delivering does]) and viability rate ([number of live kits at birth/litter size at birth] × 100).

### 2.4. Statistical Analysis

Total numbers of visible and ovulated follicles, total numbers of oocytes, and embryos were subjected to square root transformation to approximate normal distribution before subjecting to ANOVA. Data observed once a time (results of the first trial 1) including weights of different reproductive organs, numbers of ovarian follicles, ovulation points, oocytes, and embryos were analyzed by a generalized linear model (GLM) of SAS [16]. The same procedure was used for litter size weight and viability variables in the second trial. Repeated measurements including concentrations of serum LH, P_4_, and E_2_ were analyzed using the MIXED procedure of SAS. The statistical model included the fixed effect of treatment, time of sampling/data collection, and the interactions as well as the random effect of an individual female were considered. Categorical data, expressed as percentages (conception, parturition, and abortion rates) were analyzed using a chi-square test (PROC FREQ). All results were presented as least square mean ± standard error (± S.E.M.). The statistically significant differences were accepted from *p* < 0.05).

## 3. Results

### 3.1. In Vitro Assessment of the Physicochemical Properties and Release Patterns of GnRH–Loaded Nanoparticles

**Figure 3:**
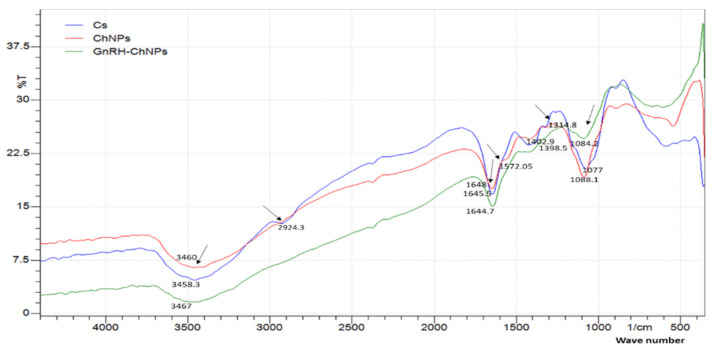
Fourier transform infrared (FTIR) spectra of chitosan (Ch), chitosan-TPP nanoparticles (ChNPs).

Physicochemical characteristics and loading efficiency of GnRH–ChNPs are shown in Table 1. The loading efficiency of GnRH by ChNPs was 90% and the average size, PdI and zeta potential of ChNPs and GnRH–ChNPs were 95.19 ± 1.9 nm vs. 212 ± 2.69 nm, 0.165 vs. 0.295, and +34.0 vs. +8.0 mV, respectively. The FTIR showed that the Ch spectra exerted peaks at 3458.3 cm^–1^, 2924.3 cm^–1^, 1645.5 cm^–1^, 1419.1 cm^–1^, 1314.8 cm^–1^, 1572.05 cm^–1^, and 1077 cm^–1^, which belong to the following functional groups: hydrogen-bond, O–H; C–H bond in pyranose rings; C=O in NHCOCH_3_; C–H in CH_2_OH; C–N stretching vibration of type ІI amine; N–H bond; and C–O–C in glucosidic-linkages. Addition of TPP to chitosan solution resulted in the disappearance of C–H and N–H functional groups, indicating binding of these groups with phosphate groups in TPP. Addition of GnRH to ChNPs resulted in shifts for the remaining functional groups identified in Ch spectra (Figure 3).

**Figure 4:**
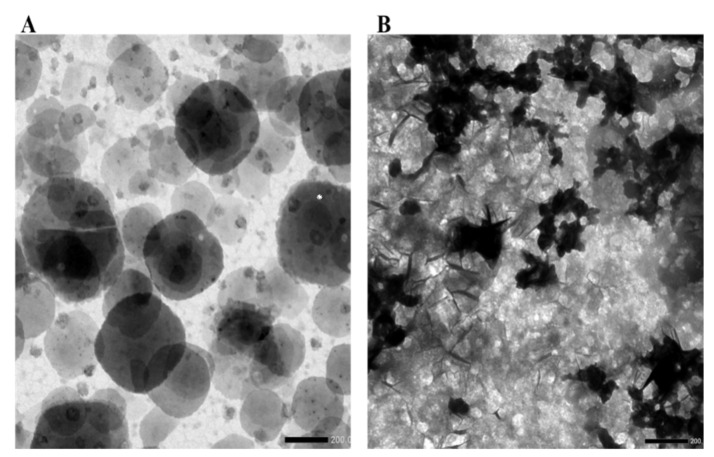
Transmission electron microscope image of (**A**) chitosan-TPP nanoparticles and (**B**) GnRH-loaded chitosan nanoparticles at a magnification × 20,000 and 25 °C.

The images of ChNPs under the transmission electron microscope showed spherical nanoparticles with smooth surfaces and a diameter of about 100 nm and, after GnRH loading, the diameter of GnRH–ChNPs reached 200 nm with high agglomerating appearance (Figure 4).

**Figure 5:**
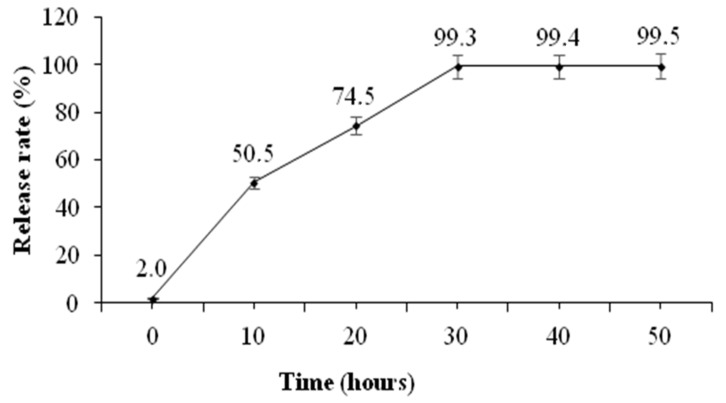
Mean (± S.E.M.) percentage of over-time release of gonadotropin-releasing hormone (GnRH) from GnRH-loaded chitosan nanoparticles.

The release profile of GnRH–ChNPs showed that the initial surge release of GnRH was about 50% at the first 10 h of incubation time, followed by a slow release of residual concentration during the subsequent 20 h. The release of the almost loaded hormone (about 99%) from ChNPs was after 30 h and remained steady up to 20 h later (Figure 5).

### 3.2. In Vivo Assessment of Patterns of LH Secretion, Ovulatory Efficiency, and Early Fertility in Does Treated with GnRH-Loaded Nanoparticles

**Figure 6:**
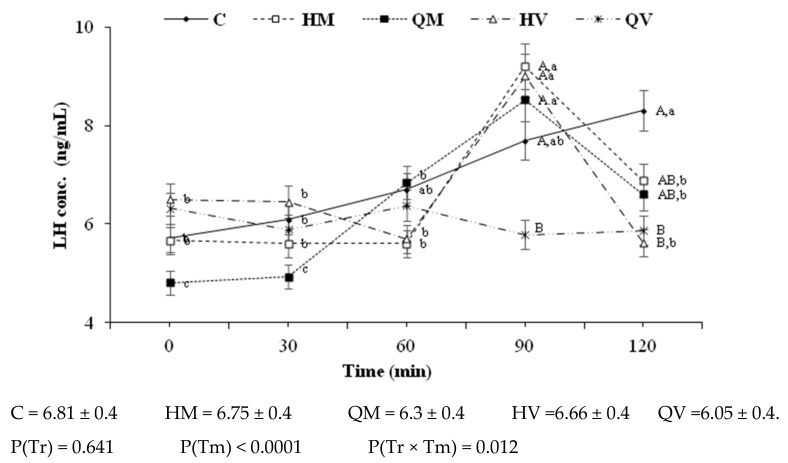
Changes over time in mean (±S.E.M.) serum LH concentrations in rabbit does receiving two i.m. doses (HM = 0.4 µg and QM = 0.2 µg) or two intravaginal doses (HV = 4 µg and QV = 2 µg) of GnRH–ChNPs or bare GnRH (C = 0.8 µg). Overall values are included below the figure. P(Tr) accounts for treatment effects, P(Tm) for time effects, and P(Tr × Tm) for their interactions. Different uppercase and lowercase superscript letters denote significant differences (*p* < 0.05) between or within treatments, respectively.

Figure 6 depicts the effect of different doses and routes of GnRH–ChNPs on serum LH concentrations at 0, 30, 60, 90, and 120 min. There were no significant effects on overall mean LH concentrations but on timing of the preovulatory LH surge. The groups HM, QM, and HV showed an earlier LH surge (90 min vs. 120 min post-insemination in the treatment C; *p* < 0.05), but the group QV failed to induce LH surge with LH concentrations remaining stable.

All groups (HM, QM, HV, and QV) significantly increased the number of total ovarian follicles when compared to group C (*p* < 0.05; Table 2). The number of total ovarian follicles was significantly lower in group HM, intermediate in groups QM and QV, and higher in group HV (*p* < 0.05). There were no significant differences in the number of ovulation points among groups, except for group QV, which showed no ovulation points.

The number of recovered embryos was higher in groups HM and HV than in group C (*p* < 0.05), while group QM showed intermediate values and no embryos were collected at group QV. The development stages of embryos recovered 48 h post-insemination from does in groups C, HM, and QM were morula and early blastocyst stages. In contrast, embryo development was advanced in group HV since 15.6 and 9.4% of the embryos were expanded blastocyst and hatching blastocyst, respectively.

### 3.3. In Vivo Assessment of Fertility and Productive Traits in Does Treated with GnRH-Loaded Nanoparticles

**Figure 7:**
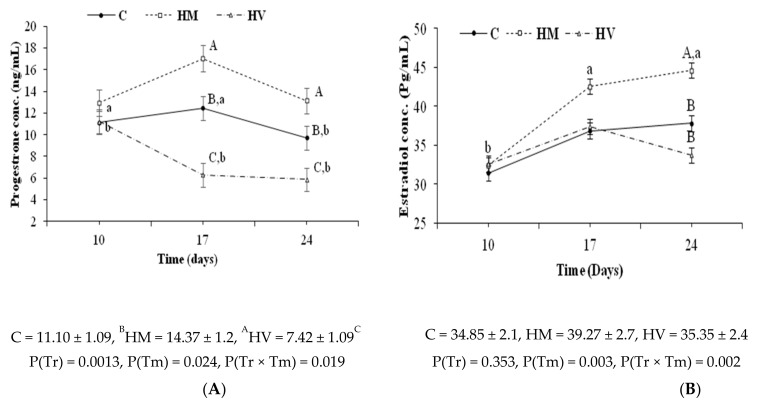
Changes over time in mean (±S.E.M.) serum progesterone (**A**) and E2 concentrations (**B**) in rabbit does receiving i.m. doses (HM = 0.4 µg) or intravaginal doses (HV = 4 µg) of GnRH–ChNPs or bare GnRH (C = 0.8 µg). Overall values are included below the figure. P(Tr) accounts for treatment effects, P(Tm) for time effects, and P(Tr × Tm) for their interactions. Different uppercase and lowercase superscripts denote significant differences (*p* < 0.05) between or within treatments, respectively.

The assessment in groups C, HM, and HV of the serum progesterone and estradiol concentrations (P_4_ and E_2_, respectively) at 10, 17, and 24 days of pregnancy (Figure 7) showed higher P_4_ concentrations in group HM than in group HV (*p* < 0.05), with intermediate values in group C. The interaction of treatment-by-time showed no differences among groups at day 10 but, from days 17 to 24, P_4_ concentrations decreased in group HV and increased in group HM (*p* < 0.05). The E_2_ concentrations were also affected by a treatment-by-time interaction (*p* < 0.05). There were no effects on days 10 and 17, but the E_2_ concentrations at day 24 were higher in group HM than in groups HV and C (*p* < 0.05). In fact, there were no changes over time in these groups whilst E_2_ concentrations increased in group HM at day 24 (*p* < 0.05).

Groups C and HM showed significantly higher conception and parturition rates than group HV and, without differences among groups in the abortion rate, also showed higher litter size, number of living litters, viability rate, and total litter body-weight at birth (*p* < 0.05; Table 3). In contrast, group HV showed a higher number of dead litter and mean body-weight at birth than groups C and HM.

## 4. Discussion

The present study aimed to set the basis for implementing a nano-drug delivery system (i.e., GnRH-loading chitosan nanoparticles; GnRH–ChNPs) for improving procedures and yields of artificial insemination (AI) in rabbits by reducing hormone dose and facilitating animal handling during AI.

Achievement of these aims depends to a far extent on the physicochemical properties of the formula. The size of NPs is one of the most important determinants for passing barriers of mucosal tissues of and for the intracellular uptake [17]. The size range of most of the nanoparticles applied for the drug delivery system is between 50–250 nm. This size allows the particles to pass efficiently through different barriers and cell pores, and thus improves the cellular uptake [18,19]. The biological efficiency of NPs greatly depends on the potential of the nano-carrier to conjugate with loaded molecules. Increasing loading efficiency of active molecules within carriers improves its lifetime in the bloodstream due to the protection from enzymatic degradation. In the present study, chitosan (Ch):TPP with a 2:1 ratio resulted in 90% loading efficiency for GnRH (Table 1). In context, Hashem and Sallam [11] obtained 91.2% LE when the same Ch:TPP ratio was used for GnRH (gonadorelin) encapsulation. On the other hand, Rather et al. [19] obtained 69% LE for LH–RH when using the same Ch:TPP ratio in female fish.; Casares-Crespo et al. [10] also obtained only 43% LE when they used a Ch:dextran ratio of 4:1 for GnRH encapsulation. These results confirm the appropriateness of selected preparation conditions and the formula (Ch concentration 1 mg/mL and Ch:TPP ratio 2:1) to conjugate most of the loaded hormone used in the present study.

In the present study, the addition of GnRH to ChNPs increased the particle size from 95.19 ± 1.9 nm to 212 ± 2.69 nm and decreased the zeta potential value from +34.0 to +8.0 mV (Table 1). Similar results have been obtained in previous studies by Kumari et al. [20], who found that the addition of trypsin increased the size of ChNPs from 147 nm to ≈220 nm. On the other hand, Hashem and Sallam [11] obtained a lower GnRH–ChNPs size after loading the 93.91 ± 0.85 nm compound to non-loaded ChNPs of 125.9 ± 3.06 nm.

The variation in the size of nanoparticles after loading (increase or decrease) could be related to many factors including the type of loaded molecules, type of carrier molecules, the preparation conditions, and the surface charge (zeta potential). Kumari et al. [20] noted that the increased diameter of ChNPs after trypsin loading might be due to the size and the molecular weight of the enzyme during the loading process and adsorption on the ChNP surface. However, in our study, the increase in GnRH–ChNP size could be ascribed to the occurrence of aggregation between GnRH–ChNPs. This suggestion is confirmed by the decreased values of zeta potential (Table 1) and image taken by transmission electronic microscope (TEM), which clearly showed the aggregation between GnRH–ChNPs (Figure 3B.). Zeta potential refers to the value of surface charge and is considered as an indicator of particle stability. Regardless of the surface charge (+ or −), high values of zeta potential (>30 mV) refer to high stability and repulsion of the particles in the solution because the high value of the electrical double-layer thickness prevents the aggregation between the particles. Thus, a low value of zeta potential accelerates the process of particle aggregation [21,22]. However, the increase in zeta potential values improves the stability of nanoparticles, this effect may not be suitable for cell viability. High zeta potential value, particularly the positive charge, stimulates cellular uptake, which affects cell survival [18,23].

The spectra of functional groups of Ch, ChNPs, and GnRH–ChNPs presented in Figure 4 were recorded in the region between 4000 cm^–1^ to 400 cm^–1^. The Ch spectra showed peaks at 3458.3 cm^–1^, 2924.3 cm^–1^, 1645.5 cm^–1^, 1419.1 cm^–1^, 1314.8 cm^–1^, 1572.05 cm^–1^, and 1077 cm^–1^, which belong to the following functional groups: hydrogen-bond, O–H; C–H bond in pyranose rings; C=O in NHCOCH_3_; C–H in CH_2_OH; C–N stretching vibration of type ІI amine; N–H band; and C–O–C in glucosidic-linkages. The addition of TPP to chitosan solution (ChNPs) resulted in the disappearance of C–H and N–H functional groups, indicating binding of these groups with phosphate groups in TPP. The addition of GnRH to ChNPs resulted in shifts for the remaining functional groups identified in the Ch spectra, which was confirmed in previous studies [19,24,25].

The results of the in vitro release study showed an initial surge release of GnRH from GnRH–ChNPs of around 50% during the first 10 h of incubation in phosphate buffer solution (PBS, pH = 7.4). This might be due to the weak bonds between adsorbed GnRH and the surface of ChNPs [26]. The other 50% of conjugated GnRH was released slowly through the incubation time (from 10 h to 30 h), which is due to the degradation of the GnRH–ChNPs’ surface and the release of encapsulated GnRH [13]. In general, nanoparticles entrap, adsorb, bind, or encapsulate the active agents in their structure, depending on the preparation method [27]. The main use of the nano-formula innovated in the present study was the induction of ovulation in rabbit does. The preparation method used in our study seems to be suitable for this purpose, since the initial release of GnRH is required to stimulate the LH surge secretion, while the slow release maintains the GnRH concentration over a long period and this sustained the surge of gonadotrophins [28].

The assessment of the potential of the developed GnRH–ChNP formula for inducing the LH-surge and ovulation revealed the relevance of the HM, QM, and HV treatments. In these three treatments, a reduction from 25% to 50% in the conventional GnRH dose was achieved without negative effects on LH surge or ovulation rate. In contrast, the QV treatment failed to induce LH surge or ovulation. These results indicate the ability of the nano-formula to protect GnRH from enzymatic degradation [20], improving the cellular uptake and thus greater bioavailability.

It is interesting to note that HV treatment advanced the pre-implanted embryo development, as 25% of the collected embryos at 48 h post-treatment were beyond the blastocyst stage whilst morula and early blastocyst were the common stages in the other groups (C, HM, and QM). In the present study, the loading efficiency of ChNPs for GnRH reached 90%. Thus, intravaginal administration of GnRH–ChNPs may result in the sustained and high release of GnRH into the reproductive tract. This suggestion is highly acceptable since it is known that the mucus layer of the reproductive tract has a surface negative charge, whereas the GnRH–ChNPs developed in our study had a positive charge (+8.0 mV). This difference in charge might increase the adhesion between the GnRH–ChNPs and the mucosal layer of the reproductive tract, allowing sustained GnRH release [11,29]. The sustained release of GnRH for a longer time may extend the ovulation time, leading to asynchrony of ova shed time. The variation in ovulation time might have resulted in the occurrence of fertilization for a longer period, and consequently a variation in embryo developmental stages. In context, it is worthy to note that GnRH is synthesized by preimplantation embryos in many species including rabbits [30]. Furthermore, GnRH has a pivotal role in the pre-implantation division and implantation of the embryo. Nam et al. [31] found that pre-implantation embryonic development could be enhanced by incubation with increasing concentration of the GnRH agonist, while GnRH antagonist had a deleterious effect. Thus, intravaginal deposition of GnRH may facilitate a GnRH binding with its receptors on preimplantation embryos, affecting their developmental stage.

Analysis of progesterone (P_4_) and estradiol (E_2_) throughout pregnancy revealed a clear reduction in P_4_ concentrations in group HV compared to groups C and HM. In rabbits, follicular and placental E_2_ is the main luteotropic factor supporting CL maintenance [15,32]. This explains the obvious increase in serum P_4_ concentration in group HM, as E_2_ concentration was the highest among groups. On the other hand, the concentrations of E_2_ showed a similar trend in groups C and HV, which indicates the implication of other factors in the maintenance of CL and P_4_ concentration. In rabbits, the pre-implantation embryo can synthesize chorionic gonadotrophins, which play a role in the maternal embryo interface [33]. The failure of this process leads to luteolysis and early embryonic loss [34]. In the present study, a proportion of the embryos, about 25%, of group HV had advanced developmental stages, while the rest of the embryos were at 48 h at the typical developmental stage. The lack of synchrony between advanced developed embryos and normally developed embryos might drive to a disturbance in the maternal-embryonic cross talking, leading to luteolysis, P_4_ reduction, and increased implantation failure and embryonic loss. Such hormonal and embryonic development disturbances may explain the lowest conception and parturition rates and litter size of group HV.

## 5. Conclusions

The fabrication of GnRH–ChNPs allows for a reduction in the conventional intramuscular GnRH dose used for AI in rabbits to half without affecting fertility. Conversely, the addition of GnRH–ChNPs to semen extenders, although successfully inducing ovulation, has negative impacts on fertility. Thus, more studies are needed to explore this point and allow further adjustments.

## Figures and Tables

**Table 1:** Particle size, polydispersity index (PdI), zeta potential, and loading efficiency of chitosan nanoparticles (ChNPs) and GnRH-loaded chitosan nanoparticles (GnRH–ChNPs).

Nanoparticles.	Particle Size (nm)	PdI	Zeta Potential (mѴ)	Loading Efficiency (%)
ChNPs	95.19 ± 1.90	0.165	+34.0	-
GnRH–ChNPs	212 ± 2.69	0.295	+8.0	90

**Table 2:** Ovarian structure and developmental stages of embryos collected 48 h post-insemination from rabbit does that received two intramuscular doses (HM = 0.4 µg and QM = 0.2 µg) or two intravaginal doses (HV = 4 µg and QV = 2 µg) of GnRH–ChNPs compared to control rabbit does that received bare GnRH (0.8 µg).

Variables	Groups (*n* = 4 Does/Group)						*p*-Value	
	C	HM	QM	HV		QV		
Ovarian structure (mean ± S.E.M.)								
Total ovarian follicles	15.5 ± 1.0 ^c^	33.0 ± 3.6 ^b^	36.5 ± 0.9 ^ab^	40.2 ± 0.2 ^a^	36.5 ± 2.0 ^ab^			0.0001
Ovulation points	6.3 ± 0.9 ^a^	9.3 ± 1.2 ^a^	6.0±1.2 ^a^	8.8 ± 1.2 ^a^	0.00 ± 0.00 ^b^			0.001
Total embryos	4.0 ± 0.7 ^b^	6.2 ± 0.9 ^a^	5.2±1.4 ^ab^	8.0 ± 0.9 ^a^	0.00 ± 0.00 ^c^			0.001
Non-fertilized ova	0.25 ± 0.3	0.00 ± 0.0	0.00 ± 0.0	0.00 ± 0.0	0.00 ± 0.00			0.438
Embryo developmental stages (%)								
Morula	50 ^b^(8/16)	48 ^b^(12/25)	76.2 ^a^(16/21)	25 ^c^(8/32)		-	0.004	
Early blastocyst	25(4/16)	40(10/25)	23.8(5/21)	50(16/32)		-	0.175	
Blastocyst	0(0/16)	12(3/25)	0(0/21)	0(0/32)		-	0.361	
Expanded blastocyst	0 ^b^(0/16)	0 ^b^(0/16)	0 ^b^(0/21)	15.6 ^a^(5/32)		-	0.009	
Hatching blastocyst	0 ^b^(0/16)	0 ^b^(0/16)	0 ^b^(0/21)	9.4 ^a^(3/32)		-	0.044	

^a, b, c^ Within rows, means with different superscripts differ at *p* < 0.05.

**Table 3:** Productive traits of rabbit does receiving intramuscular (HM = 0.4 µg) or intravaginal doses (HV = 4 µg) of GnRH–ChNPs or bare GnRH (C = 0.8 µg).

Variables	Treatments (*n* = 14 Does/Group)			*p*-Value
	C	HM	HV	
Fertility evaluation parameters^1^				
Conception rate (%)	78.5 ^a^ (11/14)	71.5 ^a^ (10/14)	50 ^b^ (7/14)	0.019
Parturition rate (%)	78.5 ^a^ (11/14)	64.2 ^a^ (9/14)	35.7 ^b^ (5/14)	0.050
Abortion rate (%)	0.0 (0/11)	10.0 (1/10)	28.5 (2/7)	0.136
Pregnancy outcomes parameters (mean ± S.E.M.)^2^				
Litter size at birth	6.08 ± 0.70 ^a^	5.47 ± 0.59 ^a^	2.50 ± 0.54 ^b^	0.001
No. of live litter	6.08 ± 0.70 ^a^	5.47 ± 0.59 ^a^	1.92 ± 0.63 ^b^	0.001
No. of dead litter	0.00 ± 0.00 ^b^	0.00 ± 0.00 ^b^	0.54 ± 0.29 ^a^	0.034
Viability rate at birth (%)	100 ^a^	100 ^a^	76.67 ^b^	0.017
Litter weight at birth (g)	60.5 ± 3.4 ^b^	66.0 ± 4.3 ^ab^	76.0 ± 6.8 ^a^	0.036
Total litter weight at birth (g)	344.2 ± 29.8 ^a^	347.33 ± 39.2 ^a^	177.3 ± 33.9 ^b^	0.003

^a, b^ Within rows, means with different superscripts differ at *p* < 0.05. ^1^ Conception rate %, (no. of pregnant does on Day 10/total no. of inseminated does × 100); parturition rate %, (no. of delivered does/no. of inseminated does × 100); and abortion rate %, (no. of aborted does/no. of pregnant doe × 100). ^2^ Litter size at birth (no. of kids at birth / no. of kindling does) and viability rate at birth % (n o. of live kids at birth/litter size at birth × 100).
